# Is enhanced cognitive behaviour therapy effective for adolescents?

**DOI:** 10.1186/2050-2974-1-S1-O58

**Published:** 2013-11-14

**Authors:** Anthea Fursland, Susan Byrne

**Affiliations:** 1 Centre for Clinical Interventions, Australia; 2 School of Psychology, University of Western Australia, Australia

## Introduction

The aim of this study was to examine the efficacy of enhanced cognitive behavioural therapy (CBT-E) for adolescents with the full range of eating disorders. There is robust evidence of the effectiveness of CBT-E for adults with eating disorders, including low weight patients. CBT-E has been adapted for adolescents and there is emerging evidence from one study (N=51) for its effectiveness in adolescents. This study aimed to add to the evidence base for the efficacy of CBT-E in adolescents with all forms of eating disorder.

## Methods

The participants (N=50, 16-18 years) were referred to a public outpatient clinic in Perth, Western Australia. 38% had a BMI <17.5. Patients attended, on average, 25 individual CBT-E sessions, plus up to 4 family sessions.

## Results

26 (52%) completed treatment. Good outcome was achieved by 61.5% of treatment completers and 32% of the total sample. Compared to those >18 years referred to this service, the adolescents were more likely to complete treatment and their outcome was similar. The results also compare favourably to those reported in the only previous case series of CBT-E with adolescents.

## Conclusion

There is encouraging evidence to support the use of CBT-E for adolescents.

This abstract was presented in the **Children and Youth Treatment and Service Development** stream of the 2013 ANZAED Conference.

